# The SW480 cell line as a model of resident and migrating colon cancer stem cells

**DOI:** 10.1016/j.isci.2024.110658

**Published:** 2024-08-05

**Authors:** Mathijs P. Verhagen, Tong Xu, Roberto Stabile, Rosalie Joosten, Francesco A. Tucci, Martin van Royen, Marco Trerotola, Saverio Alberti, Andrea Sacchetti, Riccardo Fodde

**Affiliations:** 1 Department of Pathology, Erasmus University Medical Center, Rotterdam, the Netherlands; 2 Department of Medical, Oral and Biotechnological Sciences, University of Chieti-Pescara, Chieti, Italy; 3 Department of Biomedical Sciences, University of Messina, Messina, Italy

**Keywords:** molecular biology, cell biology, cancer, transcriptomics

## Abstract

Intra-tumor heterogeneity, i.e., the presence of diverse cell types and subpopulations within tumors, presents a significant obstacle in cancer treatment due to its negative consequences for resistance to therapy and disease recurrence. However, the mechanisms that underlie intra-tumor heterogeneity and result in the plethora of different cancer cells within a single lesion remain poorly understood. Here, we leverage the SW480 cell line as a model system to investigate the molecular and functional diversity of colon cancer cells. Through a combination of fluorescence-activated cell sorting (FACS) analysis and transcriptomic profiling, we identified three distinct subpopulations, namely resident cancer stem cells (rCSCs), migratory CSCs (mCSCs), and high-relapse cells (HRCs). These subpopulations show varying Wnt signaling levels and gene expression profiles mirroring their stem-like and functional properties. Examination of publicly available spatial transcriptomic data confirms the presence of these subpopulations in patient-derived cancers and reveals their distinct spatial distribution relative to the tumor microenvironment.

## Highlights

• SW480 has 3 subpopulations with distinct cellular and molecular features
• The subpopulations are reminiscent of high-relapse, resident, and migratory CSCs
• The subpopulations have distinct spatial distributions in primary colon cancers

## Graphical abstract

**Figure:**
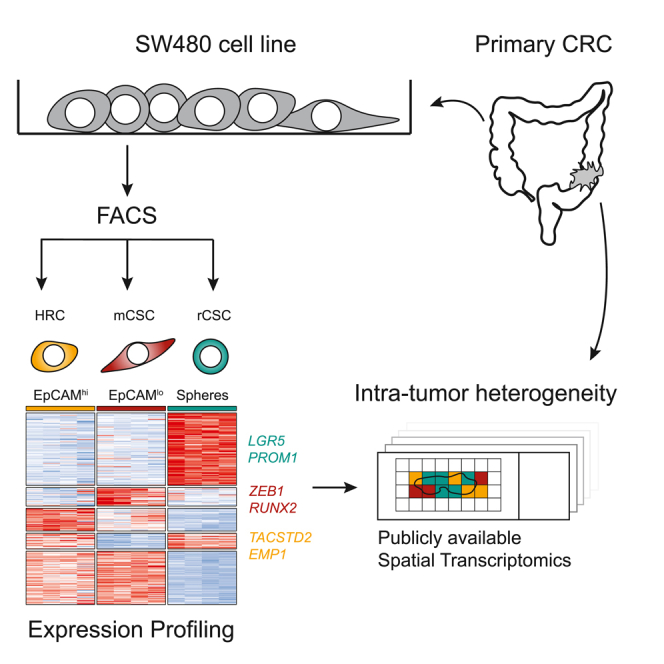

## Introduction

Intra-tumor heterogeneity is one of the main determinants of resistance to therapy and relapse.^1^ Hence, understanding the mechanisms that drive heterogeneity is crucial for our understanding of cancer. In this regard, phenotypic plasticity is increasingly recognized as the main driver of intra-tumor heterogeneity^2^ and as a major determinant of resistance to therapy, as tumor cells exploit plasticity as mechanism to adapt to changes in the environment.

In colon cancer, several studies have reported on the identification of cancer stem cells (CSC) earmarked by specific membrane-bound antigens such as *CD133*, *CD44*, and *CD166*, used to enrich for cells with tumor-propagating capacity in limiting dilution transplantation assays.^3^^,^^4^^,^^5^^,^^6^^,^^7^ These experimental approaches mainly rely on self-renewal and pluripotency rather than on their malignant and metastatic potential. Thomas Brabletz and collaborators originally postulated on the existence of a distinct CSC type, named migratory CSCs (mCSCs),^8^ usually characterized by the expression of markers such as *ZEB1* and *RUNX2*^9^ and/or by reduced expression of epithelial markers E-cadherin (*CDH1*)^10^ and EpCAM,^11^ associated with epithelial-to-mesenchymal transitions and plasticity (EMT/EMP).^12^ More recently, the *EMP1* (epithelial membrane protein 1) gene was identified as a marker for high-relapse cells (HRCs) that efficiently metastasize and correlate with poor patient outcome.^13^ Hence, colon cancer cells with metastatic capacity appear to exhibit a distinct set of markers compared to the more conventional CSCs endowed with tumor-fueling capacity.^8^

Although immortalized cancer cell lines have represented a powerful tool in cancer research, they are usually not considered equally useful as a source of CSCs. However, in 2011, Gupta and collaborators demonstrated how, even within cell lines, phenotypically distinct subpopulations of cancer cells can be found that are kept in balance through stochastic state transitions.^14^ As such, they still represent a valuable model to study phenotypic plasticity, as also shown by our previous characterization of quasi-mesenchymal mCSCs (CD44^hi^/EpCAM^lo^) in colon and ovarian cancer cell lines together with their validation in patient-derived samples.^11^^,^^15^^,^^16^

Here, we employed the SW480 cell line as a model system to study an additional subpopulation of resident colon CSC, (rCSCs, here referred to as “spheres”) thought to fuel primary tumor growth because of their enhanced self-renewal and tumor-propagating capacity.^17^^,^^18^^,^^19^ These partially adhesive, sphere-like cells are distinct from the bulk of the adherent (EpCAM^hi^) SW480 cells. By integrating data from the analysis of the SW480 sphere subpopulation and patient-derived spatial transcriptomic data, we now show that colon cancers exhibit a mixture of distinct stem-like subpopulations located within discrete and specific niches.

## Results

### SW480 encompasses three morphologically distinct subpopulations with discrete Wnt levels

**Figure 1:**
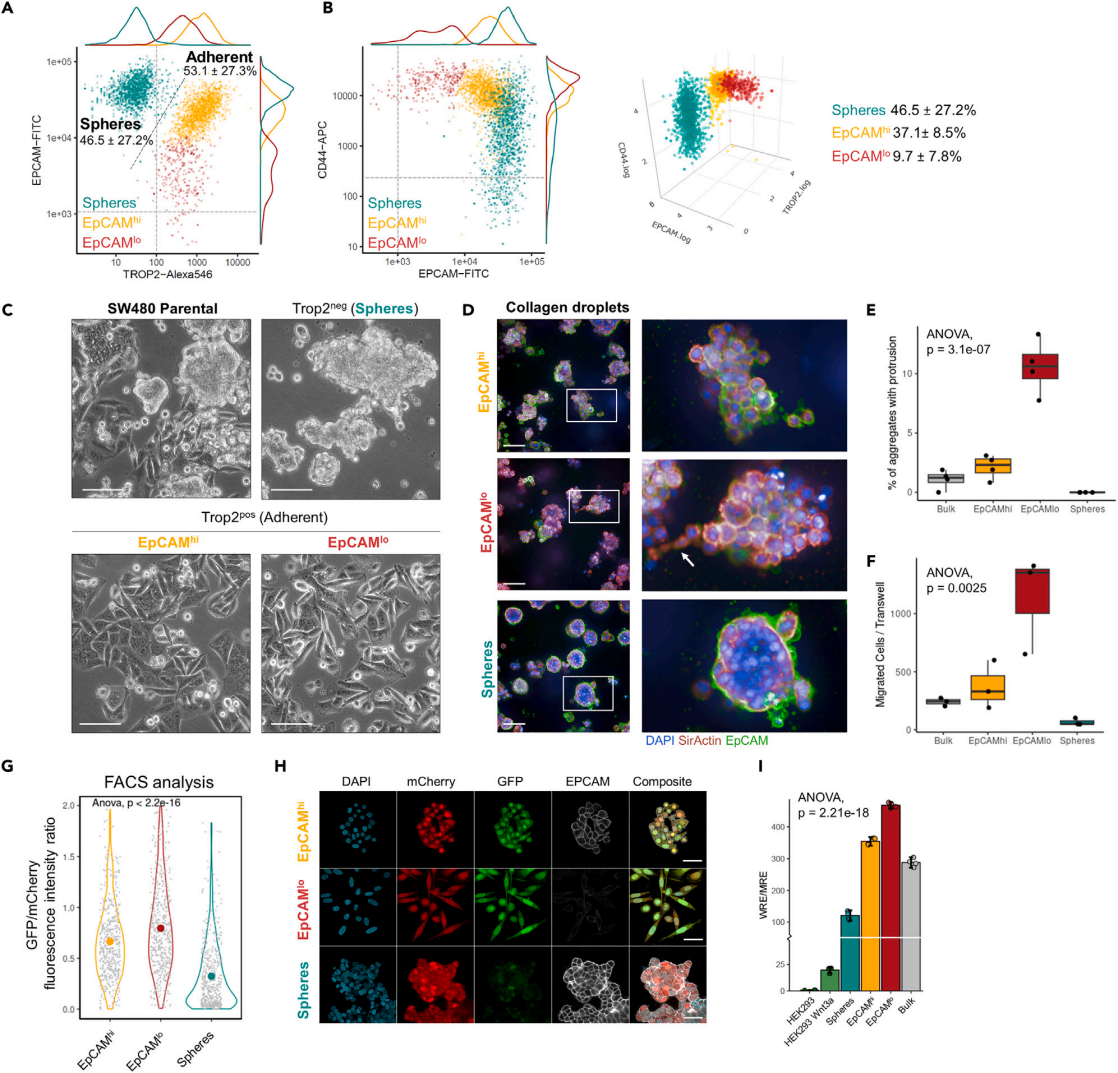
FACS-based molecular dissection of SW480 into three distinct subpopulations (A) The combination of fluorescent antibodies against EPCAM and TROP2 efficiently separates spheres (turquoise, left) from the adherent subpopulations (right) in SW480 cells. (B) Antibodies against EPCAM and CD44 can further separate EpCAM^lo^ (red) cells from EpCAM^hi^ cells (yellow) within the adherent population, and show heterogeneous CD44 levels inside the sphere population.^11^ The quadrants indicate the regions that are negative or positive for the single markers, and double-positive as shown in Figure S1. The average percentage of SW480 adherent and spheres in standard and relatively young (<1.5 months) cultures is reported in the plots. (C) Bright field pictures showing the morphology of the parental cell line and FACS-enriched subpopulations. Scale bar: 100 μm. (D) Maximum z-projections from confocal images of the collagen droplets. Representative pictures of the main morphologies observed across the distinct subpopulations. The white arrow points to a protrusion. Scale bar: 100 μm. (E) Boxplots showing percentage of cell aggregates with protrusions. (F) Boxplots denoting the number of migrated cells as measured with the *trans*-well assay. (G) Comparison of Wnt activity with the Nusse reporter by FACS. Values denote relative Wnt activity, i.e., GFP normalized by the mCherry intensity. (H) Confocal imaging of FACS-enriched subpopulations with the Nusse reporter. Scale bar: 50 μm. (I) Bar plot denoting results of TOPFlash assays for the distinct subpopulations of SW480. Data are represented as mean ± SD. *p* value denotes the result of one-way ANOVA.

We previously characterized the CD44^high^EpCAM^low^ (EpCAM^lo^) and CD44^high^EpCAM^high^ (EpCAM^hi^) subpopulations in the HCT116 and SW480 colon cancer cell lines.^11^ While doing so, we identified an additional subpopulation of EpCAM^hi^ cells in the SW480 cell line (depicted in green in Figure 1A), though not in HCT116, with a broad range (high-to-low) of CD44 expression values. Previously, these cells have been referred to as “spheres” due to their characteristic morphology, scarce adhesion to the plates, and ability to grow as tumor-spheroids in culture.^9^^,^^17^^,^^18^^,^^19^ Attempts to enrich the SW480 spheres have been implemented in various ways, ranging from the collection of floating, non-adhesive cells in culture dishes,^17^^,^^20^ or by sorting based either on their relatively low level of Wnt signaling upon transfection with fluorescent reporters,^9^ or on the increased levels of CSC markers such as CD133 (*PROM1*).^18^ Despite these efforts, a comprehensive molecular and phenotypic profile of the SW480 sphere subpopulation to allow its identification and isolation by fluorescence-activated cell sorting (FACS) is still lacking.

To this aim, we first employed a set of antibodies directed against surface (cancer) stem cell markers and adhesion molecules in combination with EpCAM and CD44. As shown in Figure 1A, the addition of an antibody against TROP2 (trophoblast cell surface antigen 2; also known as TACSTD2 or tumor-associated calcium signal transducer 2) contributed to resolve SW480 cells into three more distinct subpopulations, namely the spheres (EpCAM^hi^TROP2^neg^) and the adherent bulk, the latter to be further be subdivided in EpCAM^hi^ (CD44^hi^EpCAM^hi^TROP2^pos^) and EpCAM^lo^ (CD44^hi^EpCAM^lo^TROP2^pos^) cells (Figures 1B and S1A–S1C). In agreement with Wang et al.,^18^ CD133 expression was rarely observed in adherent cells (0.5 and 0.8% in EpCAM^lo^ and EpCAM^hi^, respectively), though it was clearly present among sphere cells (10.1%) (Figure S1D). Subsequent sorting of the three subpopulations by FACS revealed distinct morphologies, with EpCAM^hi^ cells growing in cobblestone-like colonies, EpCAM^lo^ cells as spindle-like cells, and spheres in multi-layered, compact spheroid-structures (Figure 1C).

We next evaluated the growth pattern of these cells in 3D by culturing sorted cells within collagen droplets. Of note, while sphere cells developed into compact spheroid-like structures, adherent cells exhibited a grape-like morphology with occasional protrusions (Figure 1D). The latter were most abundant in the EpCAM^lo^ collagen droplets (Figure 1E). Correspondingly, transwell assays showed that EpCAM^lo^ cells are earmarked by the highest migratory capacity, followed by EpCAM^hi^ and sphere cells (Figure 1F).

Next, we employed a lentiviral Wnt reporter based on the 7xTcf-eGFP cassette, which also ensures stable mCherry expression to distinguish infected from non-infected cells.^21^ In accordance with the Yi et al. study,^9^ sphere cells displayed lower Wnt signaling activity when compared to adherent cells, as measured by the GFP/mCherry signal ratio (Figure 1G). Among the adherent bulk, EpCAM^lo^ cells showed the highest level of Wnt signaling activity, as also confirmed by immunofluorescence (Figure 1H) and by TOP-flash reporter assay (Figure 1I). The latter confirms that, as predicted by the loss of adenomatous polyposis coli (APC) function characteristic of the SW480 cell line, all three subpopulations are Wnt-ON, albeit at variable quantitative levels. Hence, accurate isolation of three subpopulations in SW480 confirms their distinct morphologies and Wnt signaling levels.

### The three subpopulations in SW480 represent distinct transcriptional states

**Figure 2:**
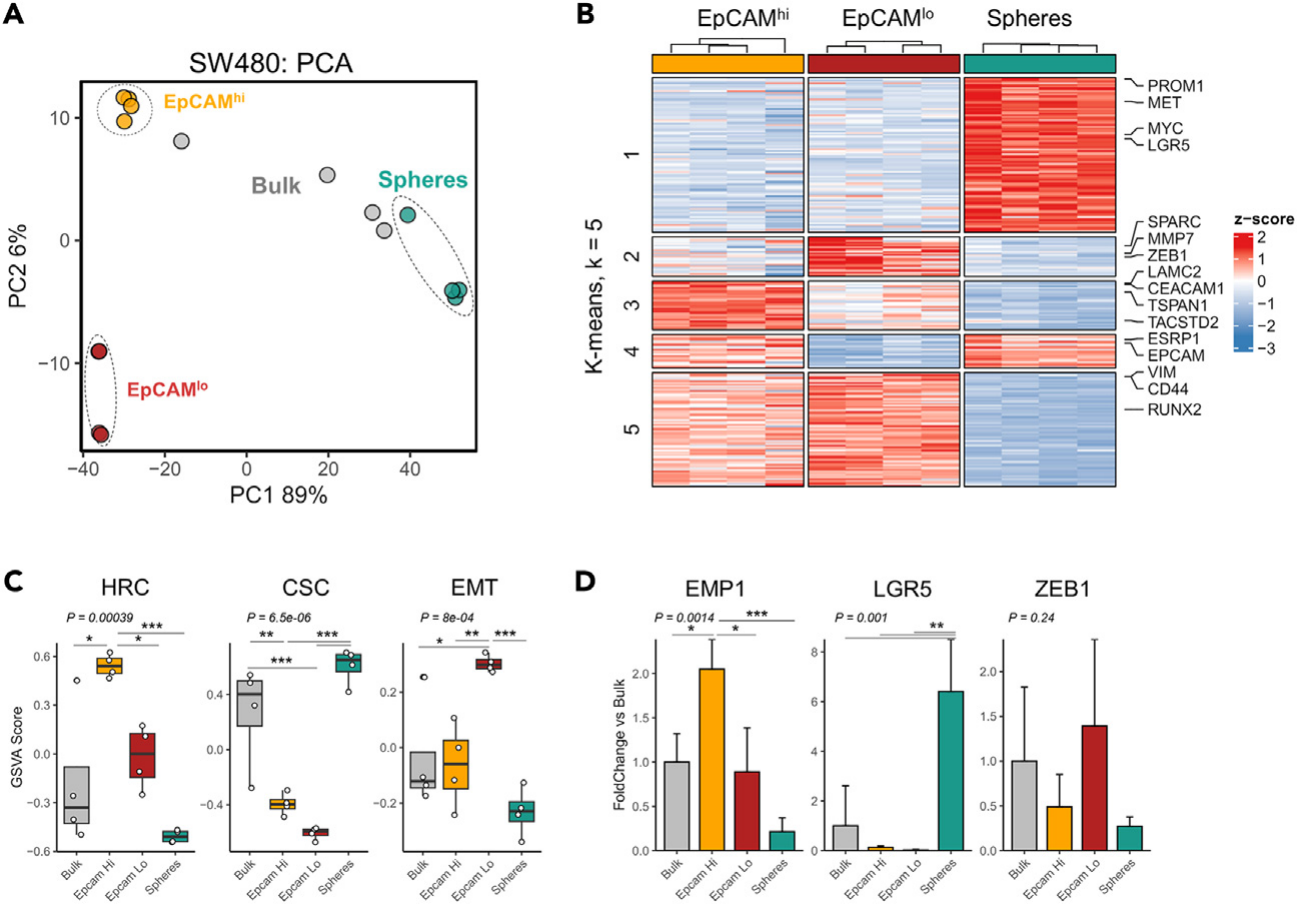
Transcriptomic analysis of the SW480 subpopulations (A) Principal component analysis (PCA) plot showing the differences between the bulk and FACS-enriched subpopulations of SW480. (B) Heatmap denoting the *Z* score normalized expression values of the differentially expressed genes. Genes were clustered into distinct groups with k-means (k = 5) clustering. (C) Evaluation of signatures for high-relapse cells (HRCs), cancer-stem cells (CSCs), and epithelial-to-mesenchymal transition (EMT) with gene set variation analysis. (D) Bar plot indicating expression level as measured by qPCR. Markers represent genes for HRC (*EMP1*), CSC (*LGR5*), and EMT (*ZEB1*). Data are represented as mean +SD. *p* value denotes the result of one-way ANOVA and Tukey’s test was performed to identify groups that differ from each other.

In order to characterize the expression profiles of the distinct SW480 colon cancer cell subpopulations, we sorted them by FACS using the aforementioned established conditions and analyzed their transcriptomes by RNA sequencing. Principal component analysis (PCA) revealed differences between spheres and adherent cells in the first principal component (accounting for 89% of the variance), while differences between the adherent EpCAM^hi/lo^ subpopulations became notable in the second principal component (6% variance) (Figure 2A). We then performed several rounds of differential expression (DE) analyses by comparing each subpopulation individually to the other subpopulations, which resulted in a total of *N* = 3,963 differentially expressed genes. Clustering of the differentially expressed genes revealed gene sets that were either expressed in two subpopulations (clusters 4 and 5 in Figure 2B), or specific to one of the subpopulations (cluster 1, spheres; cluster 2, EpCAM^lo^; and cluster 3, EpCAM^hi^; Figure 2B; Table S1). Closer examination of the gene sets revealed typical (cancer) stem cell markers in cluster 1 (spheres) such as *MET*, *PROM1* (CD133), *LGR5*, and *MYC*. The EpCAM^hi/lo^ adherent subpopulations shared gene expression patterns (cluster 5), but differed in the degree of EMT as illustrated by increased *ZEB1*, *SPARC* and *MMP7* expression in EpCAM^lo^ cluster 2. EpCAM^hi^-specific genes (cluster 3) included *CEACAM1*, *EMP1*, *LAMC2*, and *TSPAN1*, previously reported as HRC markers.^13^ Evaluation of the signatures characteristic for these identities confirmed their association with the distinct subpopulations (Figure 2C; Table S2). Additional qPCR analyses further validated expression differences in key markers that earmark the distinct subpopulations (Figure 2D).

Overall, it appears that the 3 SW480 subpopulations reflect distinct aspects of the cancer stem cell phenotype: the *LGR5*^+^ resident CSCs represented by the spheres, the EpCAM^lo^ mCSCs, and the EpCAM^hi^ HRCs. Of note, although constitutive Wnt signaling activation earmarks all 3 subpopulations, its enhanced levels in EpCAM^lo^ cells underlie EMT and the acquisition of migratory and invasive features. In this regard, whether the two metastatic subpopulations, i.e., EpCAM^lo^ and HRCs, represent distinct routes to the colonization of distant organs or are connected through stochastic state transitions is at present unclear.

### Analysis of the isotypic SW620 cell line reveals an intermediate and more homogeneous phenotype with no apparent subpopulations

**Figure 3:**
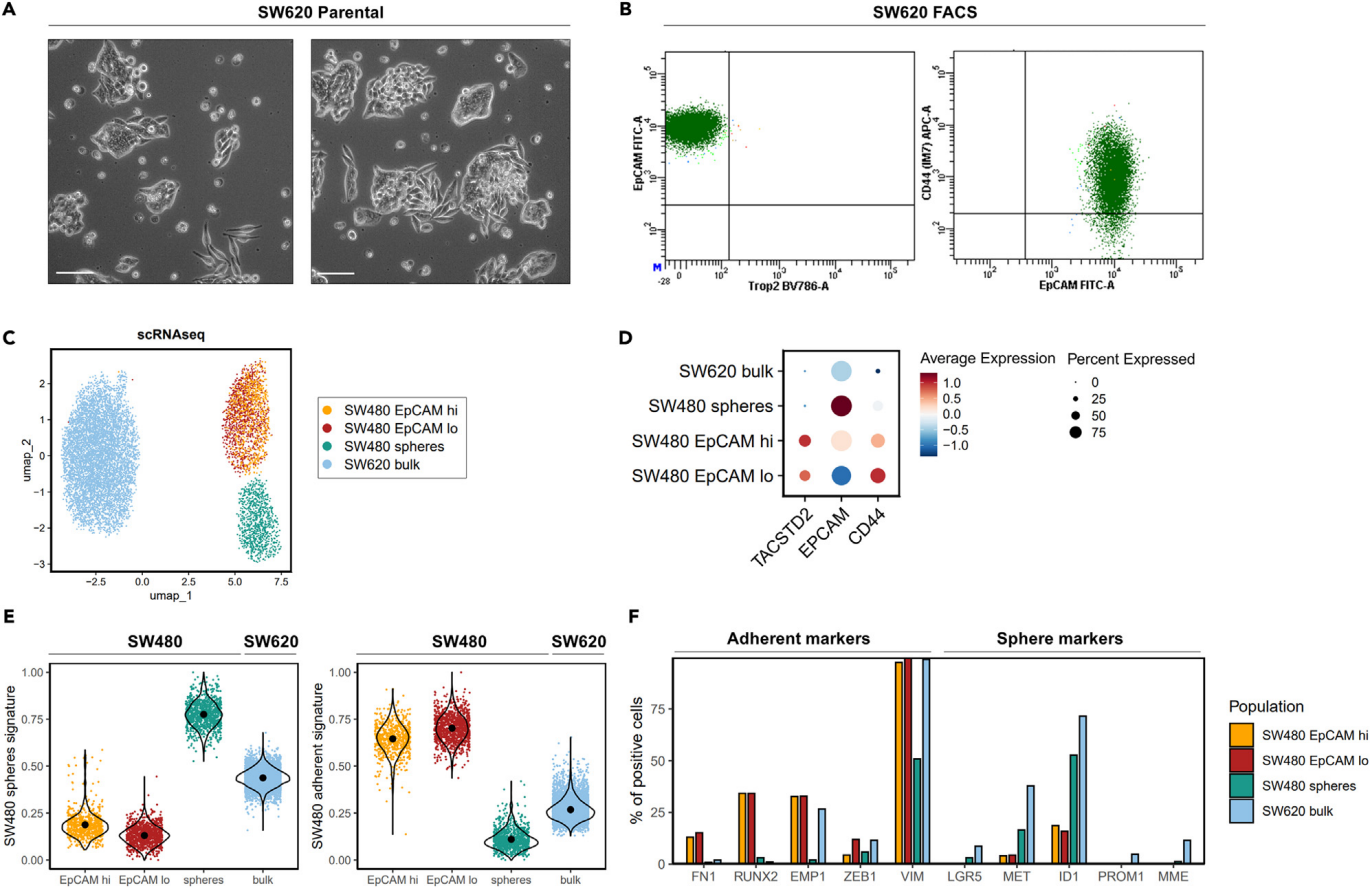
Analysis of the SW620 colon cancer cell line (A) Bright field pictures showing two examples of the morphologies observed in the SW620 parental cell line. Scale bar: 100 μm. (B) Flow cytometry analysis of SW620 cells stained for EPCAM-FITC, TROP2-BV786 and CD44-APC. (C) Uniform Manifold Approximation and Projection for Dimension Reduction (UMAP) plot showing the SW480 subpopulations and SW620 cells. (D) Dot plot indicating the average expression and percentage of cells positive for *TACSTD2*, *EPCAM*, and *CD44* across the subpopulations. (E) Violin plots showing the distribution of sphere- and adherent signatures across the distinct cell populations. (F) Bar plots denoting the percentage of positive cells for adherent and sphere markers across the distinct cell populations.

The SW620 colon cancer cell line was originally derived from a lymph node metastasis of the same tumor from which SW480 was obtained and as such represents a potentially valuable isotypic model to evaluate the subpopulations it encompasses when compared with the parental cell line.^22^^,^^23^ Notably, SW620 cells exhibited in culture both compact sphere-like cellular structures as well as more adherent, and at times spindle-like cells (Figure 3A). Flow cytometry analysis revealed that SW620 cells were TROP2 negative, similar to the SW480 spheres, and appeared more homogeneous when compared to SW480, without any distinct subpopulations when analyzed with the same EPCAM/TROP2/CD44 antibodies (Figure 3B).

Subsequent single cell RNA sequencing (scRNA-seq) analysis and dimension reduction by UMAP (Uniform Manifold Approximation and Projection for Dimension Reduction), clearly resolved the two lines, with further clustering observed between SW480 spheres and adherent cells (Figure 3C). In agreement with the flow cytometry data, SW480 spheres and SW620 were largely negative for TROP2 (*TACSTD2*), while *EPCAM* expression levels in SW620 were intermediate between to those of the SW480 EpCAM^lo^ and EpCAM^hi^ subpopulations (Figure 3D). We next generated gene signatures specific for SW480 adherent and sphere cells (Table S3) and evaluated these signatures across the different SW480 subpopulations and in SW620. Of note, SW620 exhibited an expression profile that encompassed characteristics from both the spheres and adherent cells, positioning them in between these two states (Figure 3E). This was further highlighted by the co-expression of adherent and sphere markers identified from the bulk RNA-seq (Figure 3F).

Overall, the analysis of the SW620 cell line reveals a loss of the dichotomy between sphere and adherent cells characteristic of SW480, with a more homogenous and apparently intermediate profile with markers from both subpopulations.

### Phenotypic plasticity and epigenetic barriers across the SW480 colon cancer subpopulations

**Figure 4:**
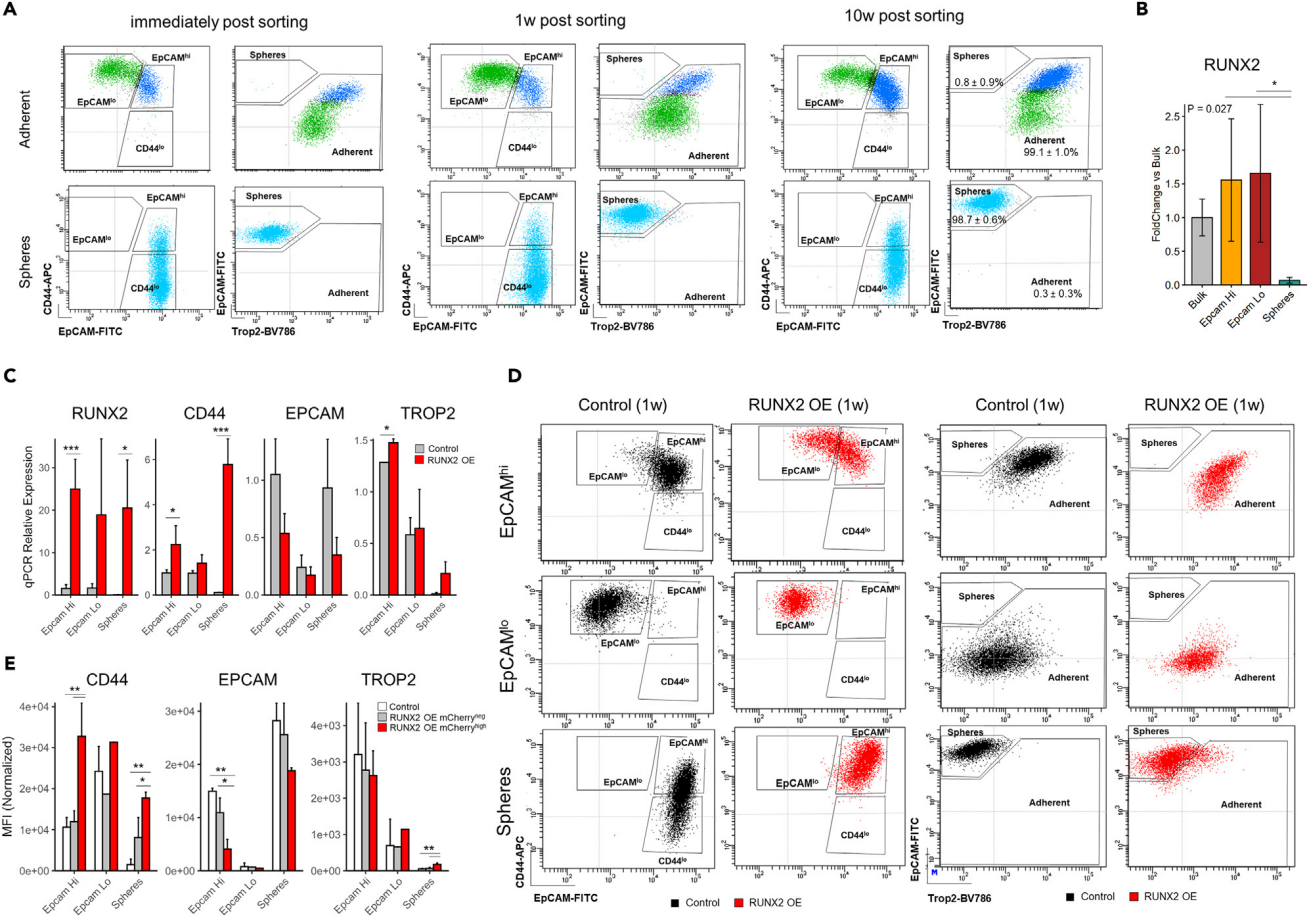
Effect of RUNX2 overexpression on the identity of SW480 subpopulations (A) Longitudinal FACS analysis of sorted adherent and sphere subpopulations. (B) Analysis by qPCR of *RUNX2* mRNA. *N* = 3 replicates. *p* value denotes the result of one-way ANOVA and Tukey’s test was performed to identify groups that differ from each other. Data are represented as mean ± SD. (C) Bar plots showing the results of qPCRs for *RUNX2*, *CD44*, *EPCAM*, and *TROP2*. Expression values were normalized against GAPDH and against the bulk control samples with the 2–ΔΔCt method. Data are represented as mean +SD. *p* value denotes the result of one-way ANOVA and Tukey’s test was performed to identify groups that differ from each other. (D) FACS analysis showing the effect of 1w overexpression of RUNX2 on the SW480 subpopulations. (E) Quantification of FACS result by comparison of mean fluorescent intensity (MFI) across different subpopulations. Gating definitions are shown in Figure S3. Data are represented as mean +SD. Asterisks denote significant levels of the adjusted *p* value after correcting for multiple testing with Tukey’s post-hoc test.

Since the EpCAM^lo^ and EpCAM^hi^ SW480 subpopulations appear to be maintained in phenotypic equilibrium by stochastic state transitions,^12^ we assessed whether the spheres also share similar plasticity and dynamics. As reported in our previous study, when sorted by FACS and subsequently cultured, EpCAM^hi/lo^ subpopulations restore the original homeostatic equilibrium after several passages.^12^ The same approach was here employed to investigate whether similar dynamics apply to the spheres and their capacity to transit toward the EpCAM^hi/lo^ subpopulations. As shown in Figure 4A, spheres were sorted and cultured for up to 10 weeks during which they retained their original cellular identity. Likewise, the adherent fraction did not give rise to sphere-like cells after prolonged culture. Hence, plasticity between SW480 adherent and sphere cells appears to be very limited under conventional culture conditions.

Next, we tested whether sphere cells can be converted by modulating the expressing of specific transcription factors known to be differentially expressed among the three subpopulations. To this aim, we first focused on RUNX2, previously shown to increase the metastatic properties of the SW480 cell line by enhancing Wnt signaling and EMT.^9^ First, we validated by RT-qPCR analysis the differential *RUNX2* expression between spheres and adherent subpopulations in the parental SW480 cell line (Figure 4B). As shown in Figure 4C, upon *RUNX2* ectopic expression, *CD44* levels dramatically increased in the spheres while a decrease in *EPCAM* is observed both in EpCAM^hi^ and sphere cells, as also confirmed by FACS (Figures 3D and S2). Of note, a significant increase in *TROP2* expression was also seen in the spheres. The sphere-specific increase in CD44 and decrease in EpCAM is indicative of a partial shift toward the adherent subpopulations upon *RUNX2* expression (Figures 4D and 4E).

**Figure 5:**
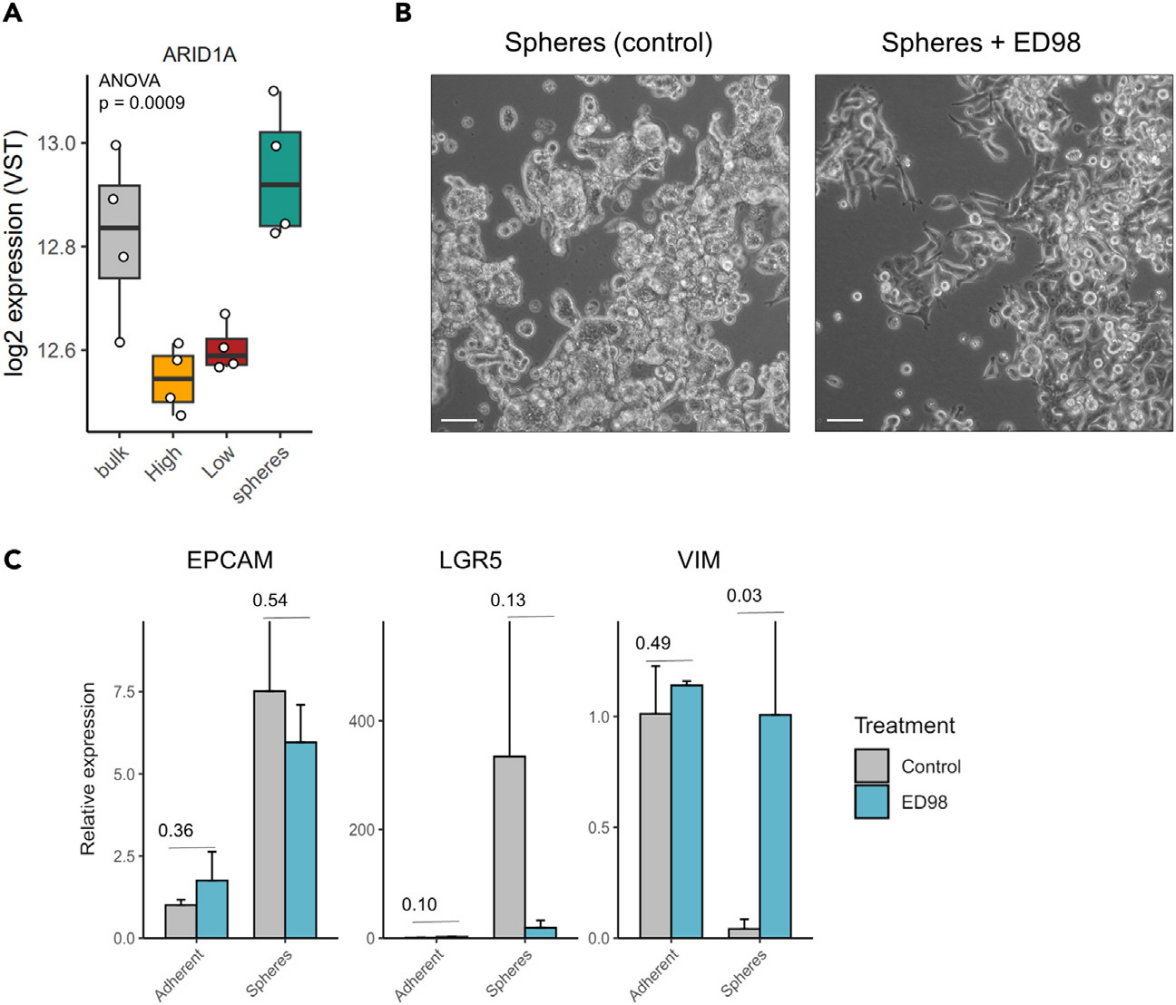
Effect of ARID1A inhibitor on the identity of SW480 spheres (A) RNA-seq expression levels of ARID1A across the distinct SW480 subpopulation. (B) Bright field images showing the effect of ARID1A inhibitor ED98 on the morphology of spheres. Scale bar: 100 μm. (C) Bar plots showing the result of ED98 treatment on the expression levels of EPCAM, LGR5, and VIM as measured with qPCR analysis. Expression values were normalized against GAPDH and against the adherent control samples with the 2–ΔΔCt method. *p* values denote significant levels of the adjusted *p* value after correcting for multiple testing with Tukey’s post-hoc test.

RNA-seq analysis of the SW480 cell subpopulations revealed a noticeable increase in *ARID1A* (AT-rich interactive domain-containing protein 1A) expression levels in the sphere cells (Figure 5A). This gene encodes for a subunit of the SWI/SNF (SWItch/Sucrose Non-Fermentable) chromatin remodeling complex. More detailed analysis of the expression of all known SWI/SNF subunits showed that, of the three broad SWI/SNF subfamilies, i.e., canonical BAF (cBAF), polybromo-associated BAF, and the GLTSCR1- or GLTSCR1L- and BRD9-containing (GBAF) complexes, cBAF appears to be upregulated in the sphere subpopulation (Figure S3). In light of these observations, we set to experimentally evaluate the effect of specific SWI/SNF perturbations on the spheres by culturing them in the presence of the ARID1A inhibitor BRD-K98645985 (from here on referred to as ED98).^24^ As shown in Figure 5B, ARID1A inhibition induced morphological changes resulting in the appearance of spindle-like adherent cells. RT-qPCR analysis of the ED98-treated spheres revealed a specific decrease in *LGR5* expression and the increase of the EMT marker *VIM* (Vimentin), whereas EpCAM did not show any significant variation (Figure 5C).

Overall, these results indicate that inhibition of ARID1A is triggers a partial conversion from spheres to adherent cells. Therefore, whereas the EpCAM^hi↔lo^ conversion seems to occur via stochastic state transitions even under normal culture conditions, the sphere-to-adherent state transition appears to be safeguarded by the SWI/SNF (cBAF) complex.

### Identification and spatial organization of the subpopulations in colon cancers

**Figure 6:**
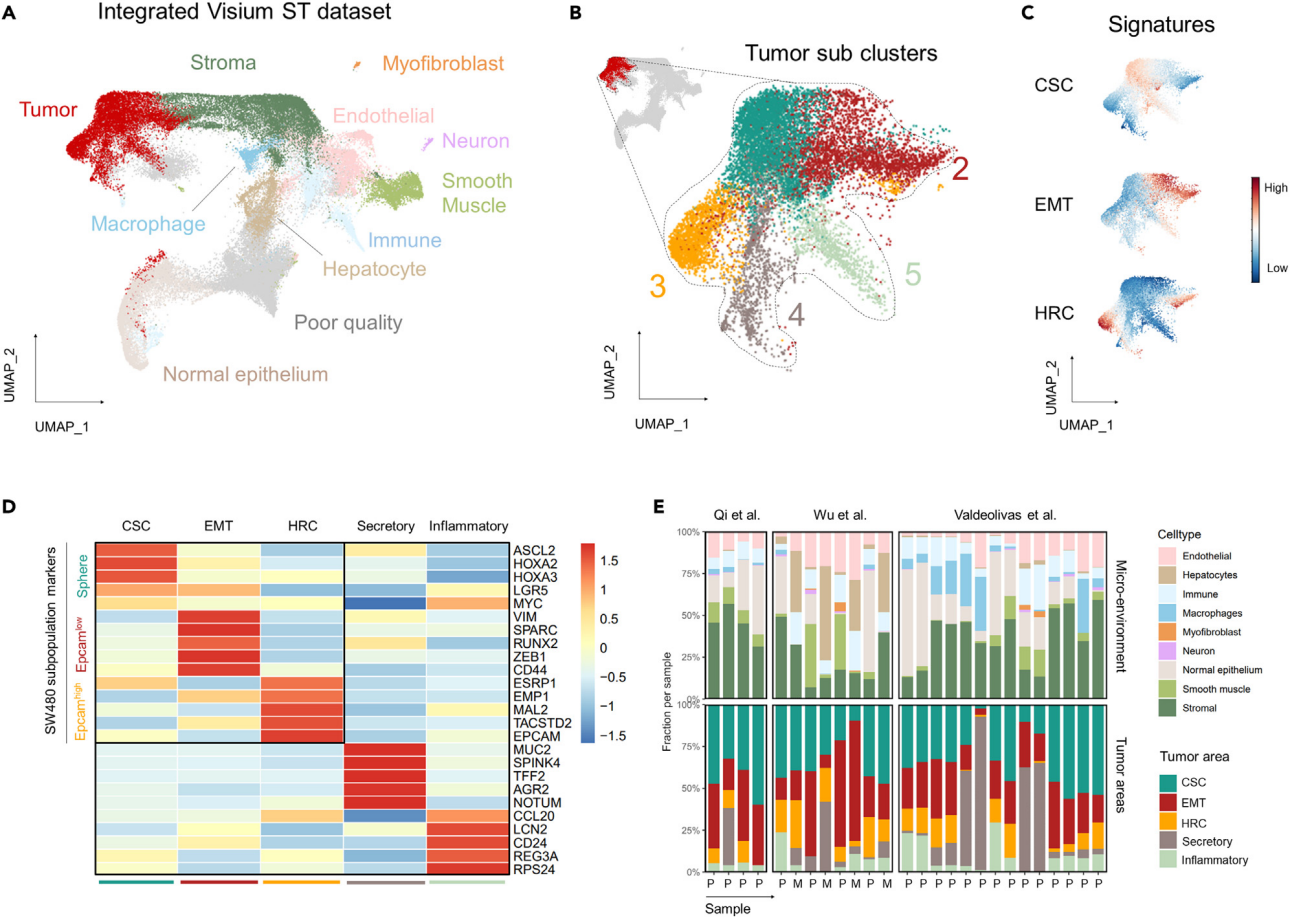
Identification of the SW480 subpopulations in primary colorectal cancer (A) Uniform Manifold Approximation and Projection (UMAP) dimension reduction plot displaying the distinct cell types across an integrated dataset of three Visium spatial transcriptomics studies. (B) UMAP plot showing the sub-clustering results of the tumor areas. Five distinct transcriptomic states were identified. (C) Evaluation of signatures for EMT, HRC, and CSC on the UMAP plot of the tumor area. (D) Heatmap showing the markers of the SW480 subpopulations, as well as additional markers for the identified ‘secretory’ and ‘inflammatory’ tumor areas. Vales denote *Z* score average expression values across the tumor sub-clusters. (E) Stacked bar plot showing the relative fraction of cell types from the micro-environment (top) and tumor entities (bottom) throughout the samples of the datasets.

To assess and validate the presence and relevance of the SW480 subpopulations in patient-derived malignancies, we interrogated a panel of spatial transcriptomic studies on colon cancers and liver metastases.^25^^,^^26^^,^^27^ We first compiled the data from the different studies resulting in a total set of 66,281 spots across 23 samples and 15 different patients (Figures S4A and S4B; Table S4). Dimension reduction and clustering resolved tumor areas from transcriptionally distinct cell types belonging to the microenvironment (Figure 6A). Subsequent sub-clustering of the tumor-specific areas revealed five tumor entities with distinct expression profiles (Figure 6B). Out of these, three showed expression profiles reminiscent of the SW480 subpopulations (Figure 5C), namely the spheres, reminiscent of resident CSCs, earmarked by expression of genes such as *ASCL2*, *LGR5*, and *MYC*; the quasi-mesenchymal EpCAM^lo^ (migrating CSCs), characterized by EMT-related markers (i.e., *VIM*, *SPARC*, and *ZEB1)*; and the EpCAM^hi^ HRCs, displaying expression of markers such as *EMP1*, *MAL2*, and *TACSTD2*. The remaining tumor entities expressed markers of secretory cells (e.g., *MUC2*, *SPINK4*, and *TFF2*) and those usually upregulated in inflammatory bowel disease (e.g., *CCL20*, *LCN2*, and *CD24*), respectively, and were accordingly labeled as “secretory” and “inflammatory” (Figure 6D). Looking at the individual tumors, the relative proportion and representation of the distinct entities appear to be variable (Figure 6E), the rCSC/sphere-like fraction being the most abundant and stable across the samples (35.9 ± 14.7%), followed by the EMT segment (28.6 ± 15.8%). The HRC subpopulation made up 10.9 ± 8.3% of the tumors, while the secretory and inflammatory areas contributed to 16.6 ± 25.4% and 8.1 ± 7.9%, respectively. No clear differences in abundance were observed between the primary and metastatic samples although, admittedly, the sample size was rather limited to discern differences between the lesions.

**Figure 7:**
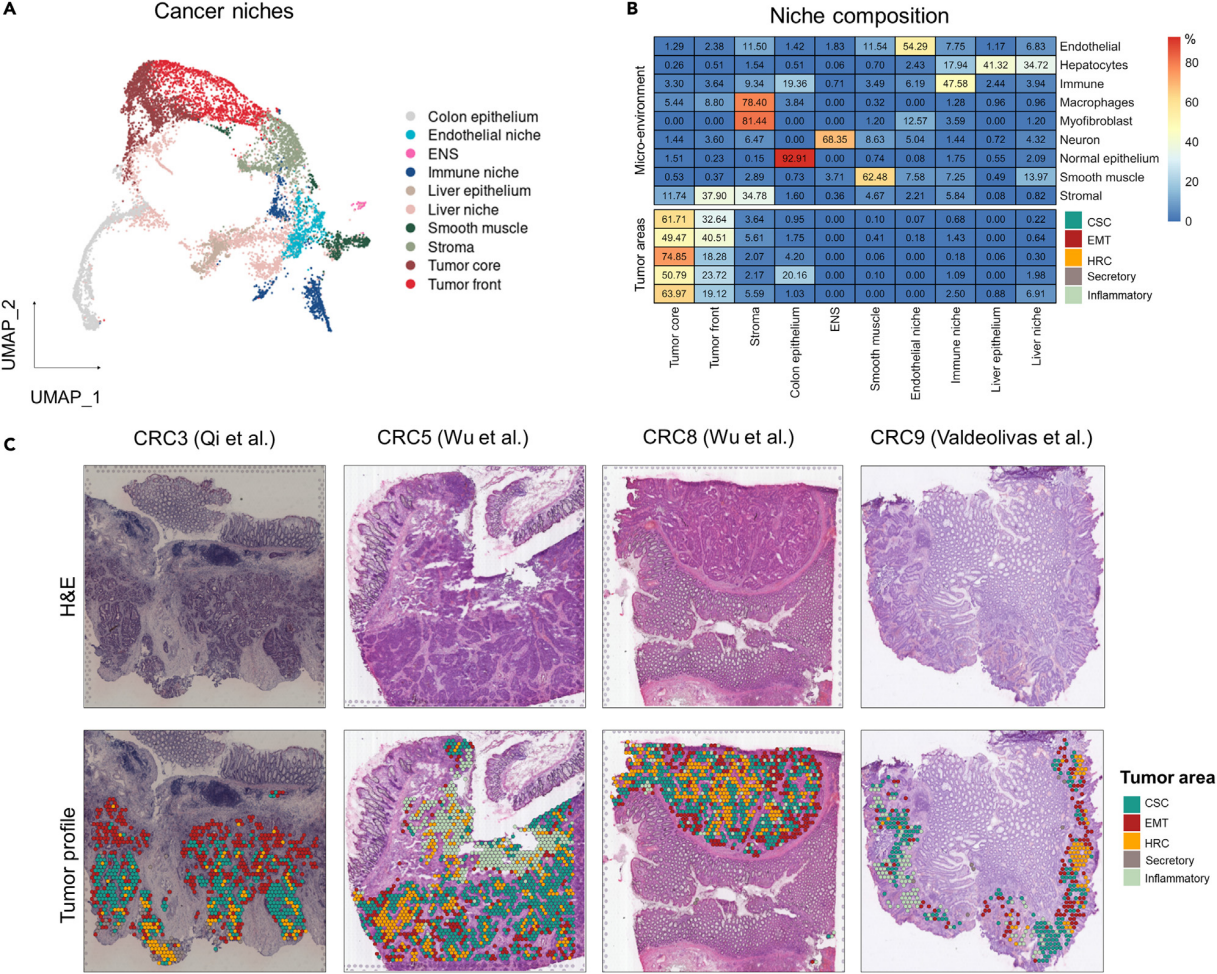
Spatial organization of tumor subpopulations in colorectal cancer (A) Uniform Manifold Approximation and Projection (UMAP) dimension reduction plot showing the expression profiles of local neighborhoods. Neighborhoods were clustered and annotated into different cancer niches. (B) Heatmap displaying the fraction of distinct cell types (rows) across the different cancer niches (columns). (C) Spatial plots showing four examples of colon cancer. Tumor areas were projected on the H&E and colored according to their transcriptomic entity.

Last, in order to investigate the spatial organization of the different cell clusters, we first grouped the data into micro-neighborhoods (see STAR Methods). We next clustered the neighborhoods based on their transcriptional profile (Figures 7A, S4C, and S4D). Using this approach, neighborhoods could be classified into different types of cancer niches, and labeled based on their composition of tumor- and stromal cells (Figure 7B). Of note, among the different tumor areas, the EpCAM^lo^-like tumor cells were most frequently observed in the tumor front (40.5% of its area). The other HRCs- and sphere-like tumor areas appeared in patches throughout the tumors. Interestingly, the sphere-like cells spatially connected distinct tumor entities, suggestive of an increased plastic potential *in vivo* when compared with the cell line (Figure 7C).

## Discussion

In this study, we outline a molecular definition by which the SW480 cell line can be dissected into three separate entities with distinct morphologies and transcriptomic states. Notably, the so-called “spheres” express a set of Wnt-related genes (e.g., *LGR5*, *MYC*, and *ASCL2*) reminiscent of the profile of the Wnt-ON intestinal stem cells in homeostasis. Somewhat counterintuitively, the Wnt signaling levels characteristic of the spheres was found to be lower when compared to the adherent, and in particular to the EpCAM^lo^ subpopulations. This observation illustrates the complex, dosage-dependent relationship between Wnt activity and its downstream effects,^28^ and underlines the role of enhanced Wnt signaling in the activation of epithelial-mesenchymal plasticity.^9^^,^^11^ The consequences of *RUNX2* overexpression in SW480 cells, i.e., increased Wnt signaling and EMT activation, underlie the partial transition from spheres into more adherent-like states.

Under normal culture conditions, the sphere “state” appears to be epigenetically fixed that prevents it from transitioning into adherent cells. This is reminiscent of intestinal crypt homeostasis where the *Lgr5*^+^ ISCs divide symmetrically and stochastically adopt stem or transient-amplifying fates following patterns of neutral drift dynamics.^29^ Our results suggest that *in vitro*, the sphere phenotype is stabilized by the expression of specific SWI/SNF subunits such as ARID1A and of transcription factors (e.g., PROX1) previously shown to reinforce an ISC-like state.^20^^,^^30^ Of note, *ARID1A* acts as a tumor suppressor gene in colon cancer and loss-of-function mutations are found in approx. 10% of the cases, often in association with the MSI-high status.^31^ Apart from somatic mutations, silencing by DNA promoter methylation was also found to be common among sporadic colon cancers.^32^ Accordingly, ARID1A inhibition by ED98 results in the partial transition from spheres to a more adherent-like morphology and expression profile.

Next, the transition from the adherent EpCAM^hi^ state into the quasi-mesenchymal EpCAM^lo^ cells is likely to be underlined by enhanced Wnt signaling and the activation of other EMT-inducing pathways. From this perspective, it is of interest to observe that both adherent SW480 subpopulations, namely the EpCAM^hi^ HRCs and the EMT-competent EpCAM^lo^ cells, have been described as highly metastatic.^11^^,^^13^ This raises the question on whether they denote different steps along the invasion-metastasis cascade, or represent two distinct metastatic routes. Additional studies are needed to clarify these issues in the near future. The present results are suggestive of a two-step sequence where HRCs may derive from resident CSCs (spheres) due to (epi)genetic perturbations of the SWI/SNF chromatin remodeling complex. Additional somatic alterations in genes leading to increased Wnt signaling such as *RUNX2* or *PROX1* will then underlie epithelial-mesenchymal plasticity and the acquisition of motile and invasive capacity.

Of note, analysis of the SW620 cell line, originally isolated from a lymph node metastasis of the same patient from which SW480 was derived,^22^^,^^23^ appears to have evolved toward an intermediate, more homogeneous state with features characteristic of both the spheres and adherent cells. Recently, it was found that two different lineage relationships between lymphatic and distant metastases exist in colorectal cancer, with the majority lymphatic and distant metastases arising from independent subclones in the primary tumor.^33^ The latter suggests that in colon cancer, the hematogenous metastatic route may be prevalent. From this perspective, it is at present not feasible to conclude whether the intermediate phenotype observed in the lymph node-derived SW620 cell line represents a highly metaplastic (and metastatic) state or a more “dormant” lineage with no relation with distant metastasis.

Admittedly, our study is centered around a single immortalized colon cancer cell line which limits its translational applicability. Nonetheless, the fact that cancer cell lines encompass distinct stem-like identities makes them an attractive *in vitro* model to study cell-autonomous mechanisms, likely to be governed *in vivo* by tumor-TME interactions, thought to be the main drivers of heterogeneity and plasticity. As shown here, interrogation of colon cancer spatial transcriptomics data from recent studies^25^^,^^26^^,^^27^ has revealed how the expression profile signatures of the SW480 subpopulations mirror distinct cellular states in clinical specimens. While these analyses are admittedly correlative, they reveal a number of cellular states recurrently observed with distinct spatial organization in patient-derived colon cancers. Whereas sphere- and EpCAM^hi^-like tumor areas appeared in patches, EpCAM^lo^-like tumor cells exhibited a more scattered pattern along the tumor margins.

Taken together, our study contributes to our understanding of cellular plasticity in colon cancer. The SW480 cell line displays an admixture of distinct cellular morphologies kept in equilibrium through stochastic state transitions driven by cell-autonomous mechanisms. In clinical specimens, the same mechanisms are regulated by interactions with the tumor microenvironment and with intra-tumor niches, as also reflected by the spatial organization of the distinct stem-like cell identities. In the near future, single-cell spatial profiling techniques will deepen our understanding of phenotypic plasticity in colon cancer and its relevance for prognosis and response to therapy.

### Limitations of the study

Clearly, the main limitation of this study lies on the fact that it is based on a single, immortalized colon cancer cell line, namely SW480. Indeed, the presence of at least 3 distinct subpopulations of colon cancer cells has been the main reason why this line was selected. Also, in a previous publication,^11^ we already have shown how one of these SW480 subpopulations, the quasi-mesenchymal EpCAM^hi^CD44^lo^, represents the phenotypic plastic cells that underlie local invasion and distant metastasis in colon cancer. As such, it appears that the SW480 molecular signatures are reminiscent of clinical colon cancer specimens.

In the present study, we extended our analysis to the so-called sphere cells, a likely proxy for resident CSCs. Clearly, it should be stated that a single immortalized cell line cannot possibly capture the complexity and heterogeneity of the carcinomas from which they are derived also in the absence of the microenvironment that is now established as a main determinant of the identity and plasticity of cancer cells. Intra-tumor heterogeneity is determined *in vivo* by a combination of cell-autonomous and paracrine mechanisms, the latter triggered from the micro-environment. Within immortalized cancer cell lines, the same mechanisms have become entirely cell-autonomous but are nonetheless worth studying as a proxy of the patient-derived carcinoma.

## STAR★Methods

### Key resources table

**Table:** 

REAGENT or RESOURCE	SOURCE	IDENTIFIER
**Antibodies**		

CD44-APC, clone IM7	BD Biosciences	RRID:AB_398661
EpCAM-FITC	GeneTex	RRID:AB_1240769
EpCAM-PerCP-Cy5.5, clone 9C4	Biolegend	RRID:AB_2098808
EpCAM-Pacific Blue, clone 9C4	Biolegend	RRID:AB_10642820
Trop2-BV786	BD Biosciences	RRID:AB_2741396
Trop2-BV510	BD Biosciences	RRID:AB_2738090
Trop2- Alexa546, clone T16	Ambrogi et al.	N/A
CD133/2-PE, clone 293C3	Miltenyi Biotec	RRID:AB_244346

**Chemicals, peptides, and recombinant proteins**		

ED98 (Baficillin)	Marian et al.^24^	N/A
Rat tail type I Collagen	Corning	#354236

**Critical commercial assays**		

Dual-Luciferase Reporter Assay System	Promega	#E1910
FuGENE HD	Promega	#E2311
Transwell	Corning	#3428
Fast SYBR™ Green Master Mix	ThermFisher	# 4385610
Single Cell 3' v2 chemistry	10X Genomics	#PN-120237

**Deposited data**		

Raw and processed RNAseq data of SW480 spheres	This paper	GEO:GSE253110
Raw and processed RNAseq data of SW480 Epcam^hi/lo^ cells	Sacchetti et al.^11^	GEO:GSE154927
Raw and processed scRNAseq data of SW620	This paper	GEO:GSE270763
Raw and processed scRNAseq data of SW480	Sacchetti et al.^11^	GEO:GSE154930
CRC Spatial Transcriptomics (Visium 10X)	Qi et al.^25^	Mendeley: ys6j8bndby
CRC Spatial Transcriptomics (Visium 10X)	Valdeolivas et al.^26^	Zenodo:7744244
CRC Spatial Transcriptomics (Visium 10X)	Wu et al.^27^	cancerdiversity.asia

**Experimental models: Cell lines**		

SW480	ATCC	RRID:CVCL_0546
SW620	ECACC	RRID:CVCL_0547

**Oligonucleotides**		

Primers for *GAPDH* FW 5′-ACCCAGAAGACTGTGGATGG-3′ RV 5′-TCTAGACGGCAGGTCAGGTC-3′	This paper	N/A
Primers for *LGR5* FW 5’- GGAAATCATGCCTTACAGAGC-3’ RV 5’- CCTGGGGAAGGTGAACACT-3’	This paper	N/A
Primers for *EPCAM* FW 5′- GCAGCTCAGGAAGAATGTG-3′ RV 5′- CAGCCAGCTTTGAGCAAATGAC-3′	This paper	N/A
Primers for *VIM1* FW 5′- GAGAACTTTGCCGTTGAAGC-3′ RV 5′- GCTTCCTGTAGGTGGCAATC-3′	This paper	N/A
Primers for *EMP1* FW 5′- GTGTTCCAGCTCTTCACCATGG -3’ RV 5′- GGAATAGCCGTGGTGATACTGC-3′	This paper	N/A
Primers for *ZEB1* FW 5′- GCACAACCAAGTGCAGAAGA -3’ RV 5′- CATTTGCAGATTGAGGCTGA -3’	This paper	N/A
Primers for *CD44* FW 5′- TACAGCATCTCTCGGACGGA -3’ RV 5′- CACCCCTGTGTTGTTTGCTG -3’	This paper	N/A
Primers for *RUNX2* FW 5′- CCCTGAACTCTGCACCAAGT -3’ RV 5′- CCCAGTTCTGAAGCACCTGA -3’	This paper	N/A
Primers for *TACSTD2* FW 5′- CGGCAGAACACGTCTCAGAA -3’ RV 5′- GCCCTGGAATAGAGACTCGC -3’	This paper	N/A

**Recombinant DNA**		

RUNX2 OE plasmid	Yi et al.^9^	Addgene #52962
7TGC plasmid	Fuerer and Nusse^21^	Addgene #24304
pMD2.G	Fuerer and Nusse^21^	Addgene #12259
psPAX2	Fuerer and Nusse^21^	Addgene #12260

**Software and algorithms**		

STAR	Dobin et al.^34^	github.com/alexdobin/STAR
DESeq2	Love et al.^35^	github.com/thelovelab/DESeq2
GSVA	Hänzelmann et al.^36^	github.com/jason-weirather/GSVA
Seurat	Hao et al.^37^	github.com/satijalab/seurat
MAGIC	Van Dijk et al.^38^	github.com/dpeerlab/magic
SF	Pebesma, E.^39^	github.com/r-spatial/sf/

### Resource availability

#### Lead contact

Further information and requests for resources and reagents should be directed to and will be fulfilled by the lead contact, Riccardo Fodde (r.fodde@erasmusmc.nl).

#### Materials availability

This study did not generate new unique reagents.

#### Data and code availability

RNA sequencing data of sorted SW480 spheres and bulk cells has been deposited in the gene expression omnibus and is publicly accessible with identifier GSE253110. Expression profiling of the EpCAM^hi^ and EpCAM^lo^ subpopulations can be accessed with identifier GSE154927. Single cell gene expression data of SW620 is accessible with identifier GSE270763 and data from SW480 can be accessed with GSE154930. The spatial transcriptomics data sets used in this study are publicly available and can be accessed from Mendely data with identifier ys6j8bndby,^25^ from Zenodo using identifier 7744244,^26^ and from cancerdiversity.asia.^27^ Microscopy data reported in this paper will be shared by the lead contact upon request. All original code has been deposited and is available at github.com/mpverhagen/iScience-24. Any additional information required to reanalyze the data reported in this paper is available from the corresponding author upon request.

### Experimental model and study participant details

#### Cell culture

The human colon cancer cell line SW480, originating from a 50-year-old white male, was obtained from the American Type Culture Collection (ATCC). SW620 was obtained from ECACC. Cell lines were cultured in DMEM medium (Thermo Fisher Scientific) with 10% FCS (Thermo Fisher Scientific), 1% penicillin/streptomycin (Thermo Fisher Scientific, #15140122), and 1% glutamine (Gibco, #25030024), in humidified atmosphere at 37°C with 5% CO_2_. The identity of the cell line and sorted subpopulations were confirmed by DNA fingerprinting with microsatellite markers (Amelogenin, CSF1PO, D13S317, D16S539, D5S818, D7S820, THO1, TPOX, vWA, D8S1179, FGA, Penta E, Penta D, D18S51, D3S1358, D21S11) and compared with the analogous data provided by ATCC, EACC, and web.expasy.org/cellosaurus (Table S5). Mycoplasma test were performed using the Mycoalert (LT07-218 Lonza) kit. Results came back negative.

### Method details

#### Collagen droplets

Cells were cultured for 3 days post sorting to recover from the FACS. Subsequently, 5k cells were plated in 30 μl droplets of pre-polymerized collagen mixture. Rat tail type I collagen (354236, Corning) was prepared at 2 mg/mL and pre-polymerized for 2-3h on ice in a mixture of collagen, 10x PBS and NaOH.^40^ After 30 min incubation at 37°C, pre-warmed medium (DMEM + 10% FCS) was added to the droplets and replaced every other day. At day 6 post seeding, droplets were washed with PBS and fixed for 3 hours in 4% PFA at 4°C. Cells were stained with DAPI, Sir-Actin (1:1000, Spirochrome, cat. no. SC001), EPCAM-FITC (1:500, GeneTex, cat no. GTX30708) and Alexa anti-mouse 488 (1:1000, Thermo Fisher Scientific, cat no. A32723). Images were acquired using the Opera Phenix HCS microscope.

#### Transwell assay

For the transwell (modified Boyden chamber) migration and assay, 5.0 × 10^4^ cells were re-suspended in DMEM medium and plated in transwell tissue culture inserts (8 μm polycarbonate membrane, 24 well, 3428; Corning). The lower compartment of the transwell chamber was filled with DMEM medium containing 10% FCS. Three technical replicates were plated for each subpopulation. The plates were incubated for 24 hours at 37°C with 5% CO2. Following incubation, cells and membranes were fixed in 4% PFA and stained with crystal violet. Non-migrated cells on the upper surface of the membrane were removed using a cotton swab, and the cells that had migrated to the lower surface of the membrane were then counted.

#### Plasmid transfection and lentiviral transduction

Stable transfection of the *RUNX2* overexpression plasmid (kind gift from Liang Fang, Addgene #52962) was performed using FuGENE HD transfection reagents (Promega, #E2311) according to the manufacturer’s protocol. Geneticin (Gibco, #10131035) was employed at a concentration of 800 μg/ml to select for transfected cells. The level of overexpression was assessed by qPCR 24h and 72h post transfection.

#### Wnt reporter assays

The 7TGC (Addgene #24304) plasmid was transfected using Fugene HD transfection reagent (Promega E2311) together with packaging vectors pMD2.G (Addgene #12259) and psPAX2 (Addgene #12260). 24 hours after transfection medium was collected and filtered. SW480 cells were transduced with the virus containing supernatant. Cells were selected based on mCherry expression. For imaging, cells were grown on glass coverslips before fixation with 4% PFA. Coverslips were counterstained with DAPI and EPCAM and imaged with a LSM700 confocal microscope (Zeiss).

For the β-catenin/TCF reporter assay (TOP-Flash reporter assay), cells were plated on 48-well dishes and cultured in DMEM medium. After 24 hrs, when 70% confluence was reached, cells were transfected by Fugene HD (Promega) with 125 ng of the TOP-Flash or FOP-Flash reporter constructs together with 25 ng of the Renilla luciferase vector for normalization purposes. As a control HEK293 cells were cultured with L-cell or Wnt-conditioned media. Luciferase activity was measured using the Dual-GLO Luciferase Reporter Assay System (Promega) 48 hrs post-transfection. Luminescence was measured using a GloMax Luminometer.

#### qRT-PCR and PCR analyses

Total RNA was isolated using TRIzol reagent (Thermo Fisher Scientific, #15596018) and was reverse-transcribed using high-capacity cDNA reverse transcription kit (Life Technologies, #4368814), according to the manufacturer's instructions. qRT-PCR was implemented using the Fast SYBR Green Master Mix (Thermo Fisher Scientific) on an Applied Biosystems StepOne Plus Real-Time Thermal Cycling Research with three replicates per group. Relative gene expression was determined by normalizing the expression of each target gene to GAPDH. Results were analyzed using the 2-(ΔΔCt) method. qRT-PCR and PCR primers are listed in the key resources table.

#### Flow cytometry and antibody staining

Cells were harvested using trypsin-EDTA (Thermofisher, #15400054), resuspended in PBS with 4% FCS, and stained on ice for 30’ with the selected panel of antibodies, each at the concentration of 5μg/ml. The following antibodies were used: CD44-APC (clone IM7, 559250, BD Pharmingen), EpCAM-FITC (ESA-214, GTX30708, GeneTex), EpCAM-PerCP-Cy5.5 (clone 9C4, 324214, Biolegend), EpCAM-Pacific Blue (clone 9C4, 324217 Biolegend), Trop2-BV786 (743277 optibuild, BD Pharmingen), and Trop2-BV510 (563244, BD Pharmingen), CD133/2-PE (clone 293C3, 130-090-853. Miltenyi),. Trop2- Alexa546 (clone T16). The latter was produced as reported in Ambrogi et al.,^41^ CD133/2-PE (clone 293C3, 130-090-853. Miltenyi) and conjugated using an Alexa Fluor™ 546 Antibody Labeling Kit (A20183. Thermofisher). After staining, cells were washed twice by centrifugation at 1200 rpm for 5’ and resuspension in PBS with 4% FCS. Flow cytometric analysis and sorting were carried out with a FACSAria III Cell Sorter (BD Biosciences, New Jersey, USA). Sequential gating on FSC-A versus FSC-W and SSC-A versus SSC-W were employed to eliminate doublets and aggregates and ensure single-cell sorting. Dead cells were excluded by gating out the fraction positive to the nuclear dye DAPI (Sigma-Aldrich, #D9542), used at 0.5 μg/mL. All the preliminary gating strategies for live/dead and doublet discrimination were performed as reported in Sacchetti et al.^11^ Additional gates, FMO samples, and compensation controls, were defined as specified in Figures S1 and S3. FITC and GFP were detected using a 488 nm laser and 502 LP and 530/30 BP filters; APC was detected with a 633 nm laser and a 660/20 BP filter; DAPI and Pacific Blue were detected using a 405 nm laser and a 450/40 BP filter; BV786 was detected with a 405 nm laser and a 750 LP and 780/60 BP filter; PE, Alexa-546 and mCherry were detected with a 461 nm laser and a 582/15 BP filter. Cell sorting was performed using a 85μm nozzle and a pressure of 45psi. Sorting purity, tested directly on the sorted fraction, was generally higher than 99.9%. To ensure absolute purity of the sorted fractions, plasticity experiments were performed with cells sorted twice.

#### Compound treatment

SW480 cells were divided into the adherent and Sphere fractions by FACS sorting. Afterwards, 25,000 cells were plated in 6-well Multi-well Cell plates with 2 mL of complete medium. The following day, the cells were treated with the following inhibitor for 48 hours: ED98 (Baficillin; ARID1A inhibitor 10uM). The cells were cultured in a 37°C, 5% CO2 incubator. 1% DMSO treated cells were also taken along as negative control. There was no effect on assay readout at DMSO. After 48hours incubation with the selected compounds, pictures were taken and RNA isolated for further characterization.

#### RNA seq analysis

TrueSeq adapter sequences were removed with Trimmomatic (v.0.33). Next, reads were aligned with STAR (v.2.4.2.a)^34^ to the human reference genome (hg38) with GENCODE (v23) annotations. A count table was generated with FeatureCounts (v.2.0.3)^42^ and downstream analysis was done in R with DESeq2 (1.36.0).^35^ Counts were normalized with variance stabilizing transformation (VST) and principal component analysis (PCA) was performed on the top500 variably expressed features. Differential expression (DE) analysis was done by comparing each subpopulation to another using a Wald test with Benjamini-Hochberg adjustments for multiple testing. DE genes were selected based on p_adj < 0.05 and log2FoldChange > 1.5. For heat map visualization, DE genes were z-score normalized and clustered with k-means (k = 5). Signatures were evaluated with a gene set enrichment analysis (GSVA, v1.44.5)^36^ and GSVA scores were compared with ANOVA to test for significance.

#### Single cell RNA sequencing

Cell lines were cultured to 60–70% confluency before initiating the experiment. For each sample, between 5×10^4^ and 1×10^5^ cells were sorted by FACS and processed with the 10x Genomics Chromium Single Cell Controller (Single Cell 3' v2 chemistry). Samples underwent deep sequencing on an Illumina platform (HiSeq 2000), achieving a depth of 50k reads per cell. Gene-cell matrices were generated by aligning the reads to the human transcriptome (GRCh38) using the Cell Ranger pipeline (v2.1.1). The filtered gene-cell matrices were then merged in R and subjected to downstream analysis using the Seurat package (v4.3.0).^37^

#### Analysis of spatial transcriptomics data

Pre-processed 10X Visium data sets^24^^,^^25^^,^^26^ were imported in R and analyzed with Seurat (v4.3.0).^42^ Batch correction was performed with 2000 integration anchors using the reciprocal PCA (RPCA) method. Next, dimension reduction was performed with Uniform Manifold Approximation and Projection (UMAP; min.dist = 0.2, n.neighbors = 100, spread = 2) based on the first 50 principal components. Unsupervised clustering was done with the Leiden method (resolution = 0.2). Clusters of spots were annotated according to the expression of marker genes and by comparison with the previously published annotations. Tumor spots were sub-clustered with FindSubCluster (Louvain algorithm, resolution = 0.25), and annotated according to the expression of markers from the SW480 subpopulations. Signature scores were derived by averaging the Markov Affinity-based Graph Imputation of Cells (MAGIC)^38^ imputed values after a z-score normalization to equalize gene weights. Neighborhood analysis was done with the SF package^39^ by aggregating the expression profile of neighboring spots in honeycomb grids of 4x4 to create local niches. Subsequently, UMAP dimension reduction was performed and niches were clustered with the Leiden method (resolution = 0.3). Niches were annotation according to the composition of respective cell types. Spatial patterns were visualized with the SpatialDimPlot function in Seurat.

### Quantification and Statistical analysis

Statistical tests were performed in R (version 4.3.2). Two compare whether significant differences were present across multiple groups, analysis of variance (ANOVA) was performed. For multiple group testing, p value after correcting for multiple testing with Tukey post-hoc test.

## Supplemental information

Document S1. Figures S1–S4

Table S1. Gene groups defining SW480 subpopulations, related to Figure 2B

Table S2. Gene signatures employed in this study, related to Figure 2C

Table S3. Differential expression analysis of single cell data, related to Figure 3

Table S4. Meta data of spatial transcriptomics cohort, related to Figure 6

Table S5. Short tandem repeat analysis the SW480 and SW620 cell line, related to STAR Methods
