# Supplementary material for: The SW480 cell line as a model of resident and migrating colon cancer stem cells
Source: iScience. 2024 Aug 5;27(9):110658. doi: 10.1016/j.isci.2024.110658 (PMC11379671; doi:10.1016/j.isci.2024.110658)
Supplement: Document S1. Figures S1–S4 [file mmc1.pdf]

iScience, Volume *27*

Supplemental information

The SW480 cell line as a model of resident and migrating colon cancer stem cells

Mathijs P. Verhagen, Tong Xu, Roberto Stabile, Rosalie Joosten, Francesco A. Tucci, Martin van Royen, Marco Trerotola, Saverio Alberti, Andrea Sacchetti, and Riccardo Fodde

Supplemental information

Document S1. Figures S1–S4 and Tables S1-S5

Figure S1.	Staining and compensation controls relative to Figure 1.
Figure S2.	FACS Staining and compensation controls relative to Figure 4.
Figure S3.	Expression of SWI/SNF complex subunits across the different subpopulations relative to Figure 5.
Figure S4.	UMAP embedding before and after batch-correction, related to Figure 6 and 7.

Table S1.	Gene groups defining SW480 subpopulations, related to Figure 2B.
Table S2.	Gene signatures employed in this study, related to Figure 2C.
Table S3.	Differential expression analysis of single cell data, related to Figure 3
Table S4.	Meta data of spatial transcriptomics cohort, related to Figure 6.
Table S5.	Short tandem repeat analysis the SW480 and SW620 cell line, related to STAR Methods.

Figure S1

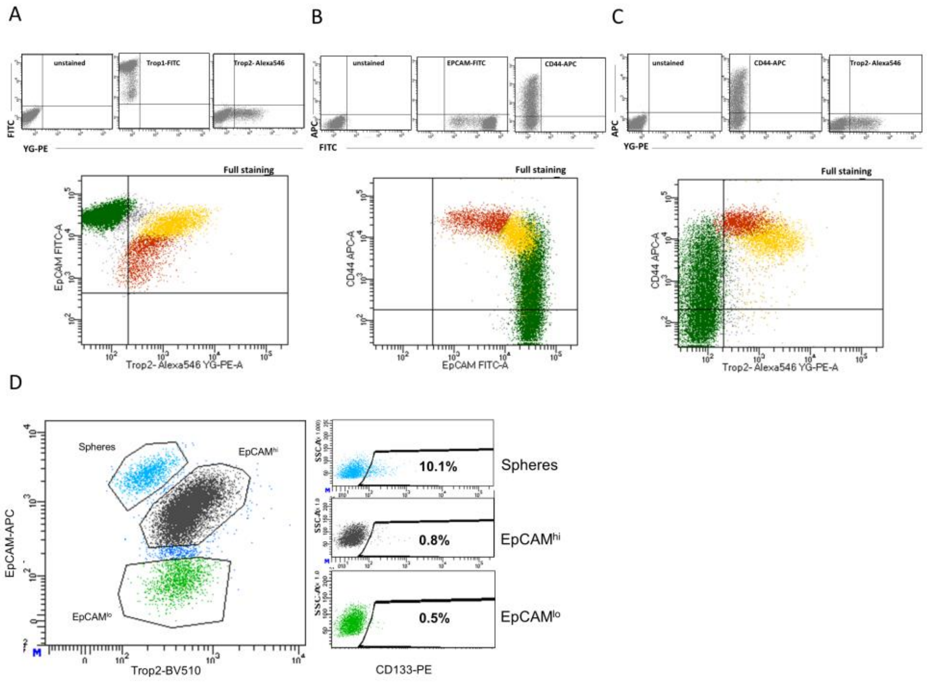

Figure S1. Staining and compensation controls relative to Figure 1.

(A) EpCAM FITC vs Trop2-BV546

(B) EpCAM FITC vs CD44-APC

(C) CD44-APC vs Trop2 Alexa546. Unstained = mix of isotype controls.

(D) FACS plot showing the three different subpopulations in SW480 (left) with their corresponding expression of CD133 (right).

Figure S2

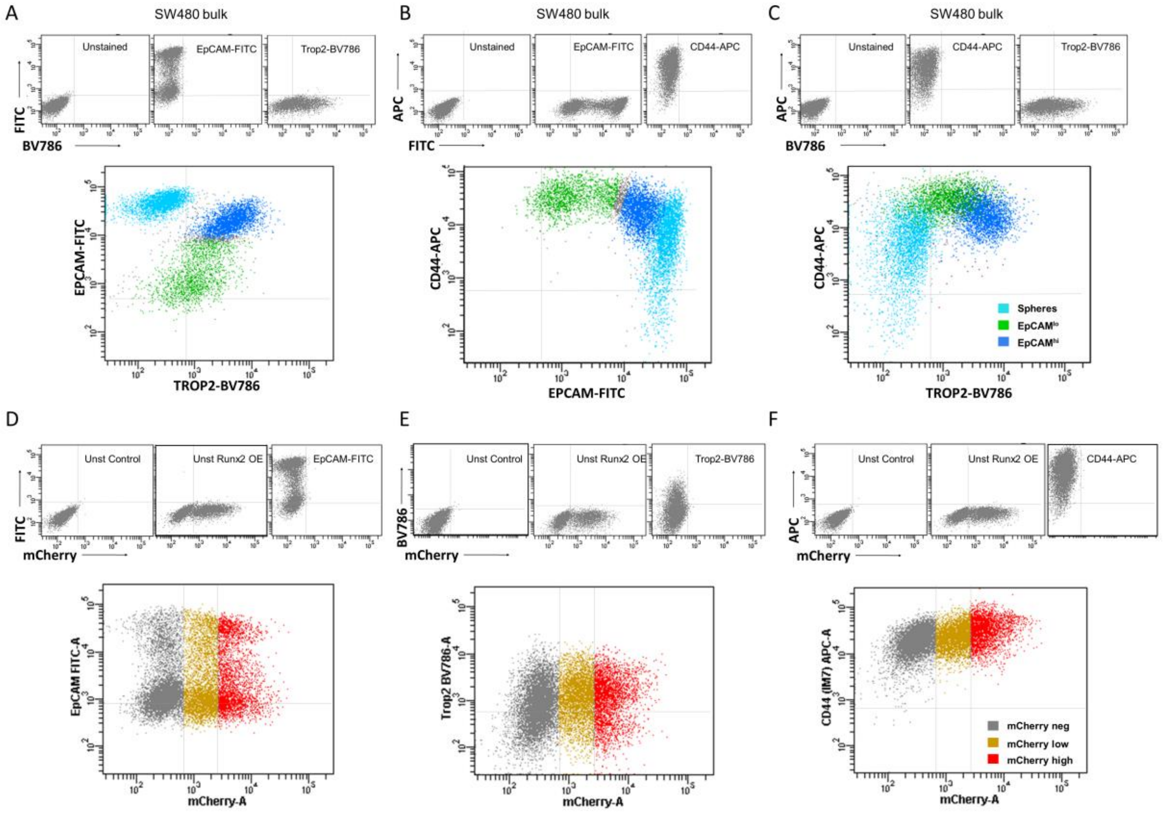

Figure S2. FACS Staining and compensation controls relative to Figure 4.

(A) EPCAM vs. TROP2. Related to Figure 4A.

(B) CD44 vs. EPCAM. Related to Figure 4A.

(C) CD44 vs. TROP2. Related to Figure 4A.

(D) Plots relative to bulk SW480/RUNX2. EPCAM vs. the mCherry/RUNX2 reporter. Related to Figure 4D.

(E) TROP2 vs. the mCherry/RUNX2 reporter. Related to Figure 4D.

(F) CD44 vs. the mCherry/RUNX2 reporter. Related to Figure 4D.

Figure S3

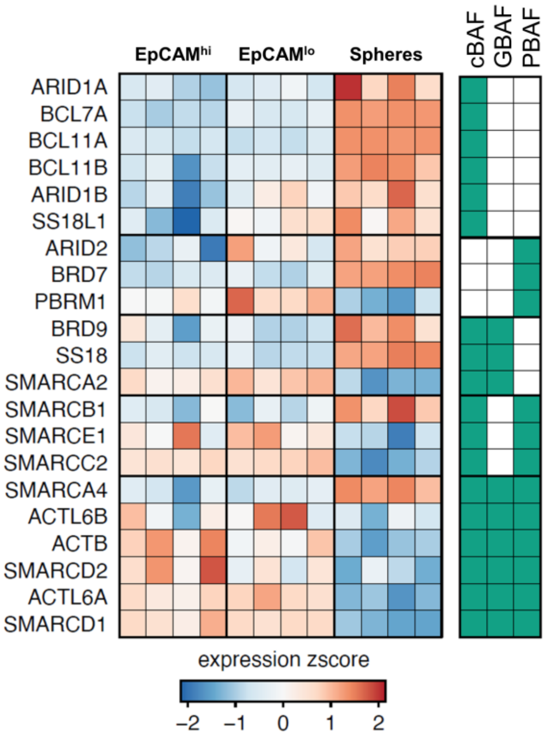

Figure S3. Expression of SWI/SNF complex subunits across the different subpopulations relative to Figure 5.

Heat map denoting the z-score normalized expression value of the RNAseq data.

Figure S4

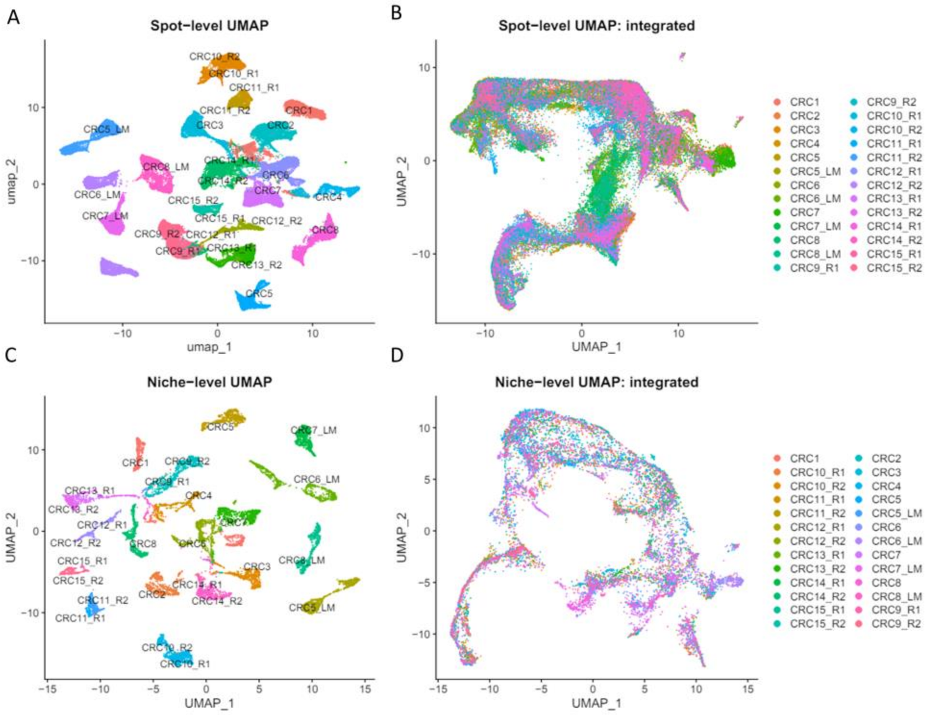

Figure S4. UMAP embedding before and after batch-correction.

(A) UMAP plot showing merged data set on the Visium spot level. Related to Figure 6.

(B) UMAP plot after integration with the reciprocal PCA (RPCA) method. Related to Figure 6.

(C) UMAP plot showing merged data set on neighborhood level. Related to Figure 7.

(D) UMAP plot of the neighborhoods after integration with the reciprocal PCA (RPCA) method. Related to Figure 7.
